# Dietary cholesterol intake and stroke risk: a meta-analysis

**DOI:** 10.18632/oncotarget.23933

**Published:** 2018-01-04

**Authors:** Pengfei Cheng, Junxi Pan, Jinjun Xia, Fengli Deng, Wen Huang, Shunjie Bai, Xiaofeng Zhu, Weihua Shao, Haiyang Wang, Peng Xie

**Affiliations:** ^1^ Department of Neurology, The First Affiliated Hospital of Chongqing Medical University, Chongqing, 400016, China; ^2^ Chongqing Key Laboratory of Neurobiology, Chongqing, 400016, China; ^3^ Institute of Neuroscience and The Collaborative Innovation Center for Brain Science, Chongqing Medical University, Chongqing, 400016, China; ^4^ Department of Neurology, The First Affiliated Hospital of Jiamusi University, Jiamusi, Heilongjiang Province, 154002, China; ^5^ The M.O.E. Key Laboratory of Laboratory Medical Diagnostics, The College of Laboratory Medicine, Chongqing Medical University, Chongqing, 400016, China; ^6^ Department of Neurology, Xinqiao Hospital, Third Military Medical University, Chongqing 400037, China; ^7^ Institute of Neuroscience, Jiamusi University, Jiamusi, Heilongjiang Province, 154002, China; ^8^ Department of Respiratory Medicine, The First Affiliated Hospital, Chongqing Medical University, Chongqing, 400016, China

**Keywords:** diet, cholesterol, stroke, cerebrovascular accident, meta-analysis

## Abstract

**Background/Objectives:**

The association between dietary cholesterol and stroke risk has remained controversial over the past two decades. The aim of this meta-analysis was to assess the relationship between dietary cholesterol and stroke risk.

**Results:**

Seven prospective studies including 269,777 non-overlapping individuals (4,604 strokes) were included. The combined RR of stroke for higher cholesterol intake (> 300 mg/day) was 0.98 (95% CI, 0.90–1.07), and the combined RR of stroke for higher cholesterol intake (> 300 mg/day) in females (age of ≥ 60 years or body mass index of ≥ 24 kg/m^2^) was 1.18 (95% CI, 1.02–1.36).

**Materials and Methods:**

The PubMed, Medline, Embase, Web of Knowledge, and Google Scholar databases were searched. Relevant studies were identified by searching these online databases through September 2017. The relative risk (RR) and 95% confidence interval (CI) were used to investigate the strength of the association.

**Conclusions:**

Higher cholesterol intake has no association with the overall stroke risk. Age and body mass index affect the relationship between dietary cholesterol intake and stroke risk. However, the association between higher dietary cholesterol and stroke risk in males remains unclear.

## INTRODUCTION

Stroke, also known as cerebrovascular accident, is the third leading cause of death and the main cause of disability in both developed and developing countries worldwide [1–3]. Stroke results in many complications [4, 5] and caused an estimated 5.5 million deaths annually [6]. Approximately 15 million people develop a stroke every year according to World Health Organization reports, and stroke has become a great health burden in recent decades [7–9]. Stroke sur*vivo*rs are more frequently disabled and have a higher risk of dementia than stroke-free individuals [10]. High dietary cholesterol increases the degree of lipid peroxidation, which is related to the development of atherosclerosis [11]. Some evidence [12] has shown that dietary cholesterol intake has a positive relationship with blood pressure, which is a crucial risk factor for stroke [13–15]. The association between dietary cholesterol intake and stroke risk has been examined over the past 20 years. However, these studies reported cholesterol intake using different measurement units (e.g., grams, servings, or tertiles, quartiles, and quintiles), and grams of cholesterol intake varied among the studies. Therefore, evidence of a relationship between a certain cholesterol intake in grams and stroke risk is lacking. Evidence of a relationship between dietary cholesterol intake and the risks of different types of stroke (ischemic or hemorrhagic) is also lacking. Moreover, whether the body mass index (BMI) influences the relationship between higher cholesterol intake and stroke risk remains unknown. Therefore, the association between cholesterol intake and the risk of stroke must be clarified to formulate efficient preventive strategies.

We performed a systematic review and meta-analysis of cholesterol intake and stroke risk to identify the associations between (i) cholesterol intake and stroke risk based on the epidemiologic characteristics of the study population and (ii) the dietary cholesterol intake and the risk of stroke according to stroke subtype.

## RESULTS

**Figure 1:**
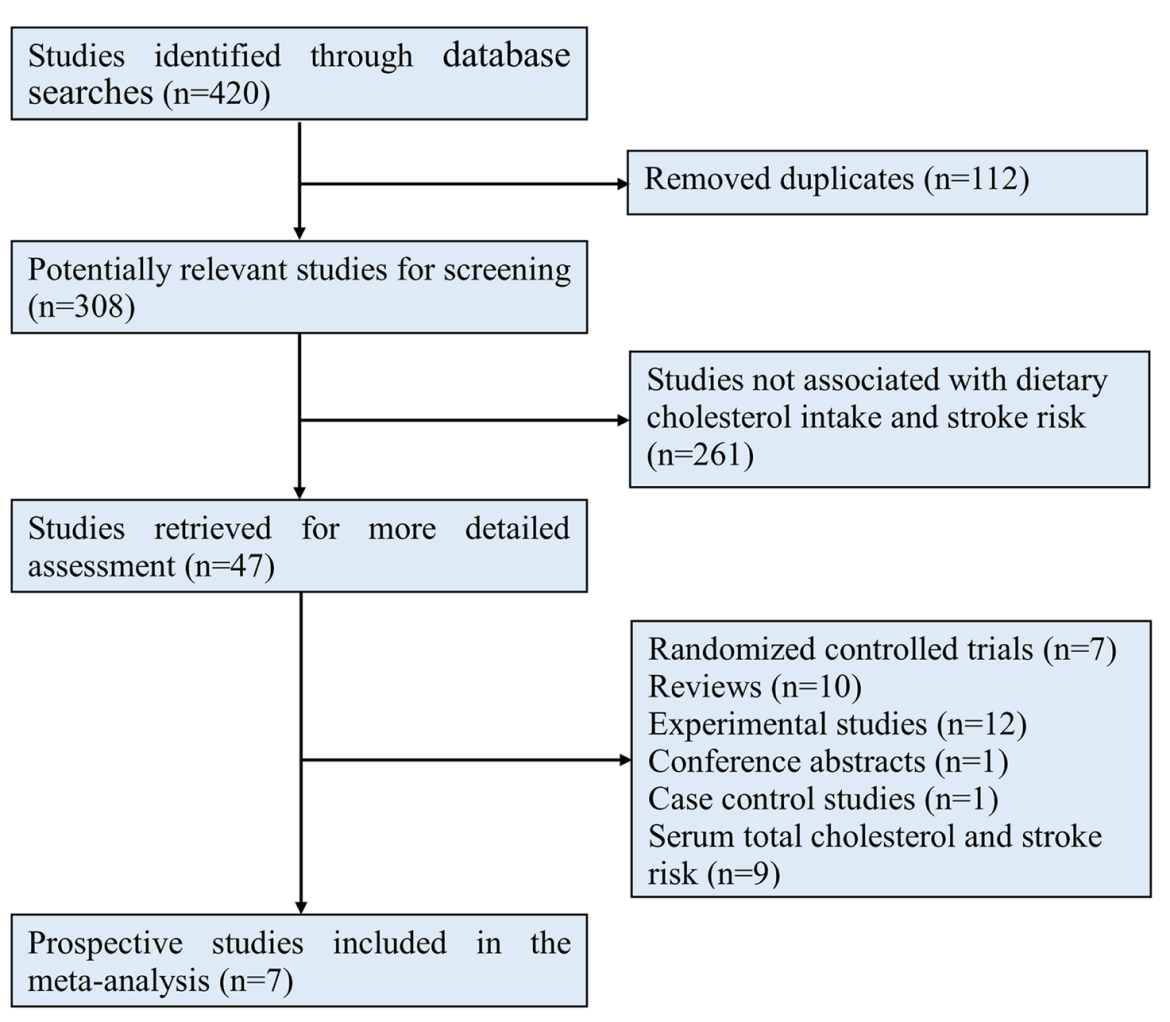
Flow chart of studies selection

The search strategy revealed 420 citations (Figure 1). After screening of the titles and abstracts, 47 full-text articles were evaluated. After the final exclusions, 7 original studies [16–22] were included in this meta-analysis, comprising 269,777 individuals and 4,604 stroke events (Figure 1). The baseline characteristics are shown in Table 1. The studies included in this meta-analysis were published from 1997 to 2012. Five studies [16, 18, 19, 21, 22] used the Food Frequency Questionnaire, which was validated by the investigators. Two studies [17, 20] used 24-hour recall to assess the dietary cholesterol intake, and both of these studies confirmed the reproducibility of the 24-hour recall data. The follow-up duration ranged from 7.6 to 15.5 years. The number of participants ranged from 2,283 to 87,025, and the number of stroke events ranged from 75 to 1,680. Three studies [17, 20, 21] were from Japan, three studies [16, 18, 22] were from the United States, and one study [19] was from Sweden. The study quality scores are shown in Supplementary Table 1.

**Table 1:** Baseline characteristics of included studies

First author	Year	Age range (mean age)	Sex	No. of Participants	Cholesterol intake assessment	Average BMI	Average Follow-up, yr	Stroke Events	Country	Fatal or non-fatal strokes	Maximum adjustment available
Seino	1997	40–89	Both	2,283	FFQ	< 24	15.5	75	Japan	Both	Age, sex, DBP, atrial fibrillation, and total energy intake.
Iso	2001	34–59 (< 60)	Female	85,764	FFQ	NA	14	690	USA	Both	Age, smoking, time interval, BMI, alcohol, menopausal status, hormone use, exercise, aspirin use, multivitamin use, vitamin E use, n-3 fatty acid intake, calcium intake, hypertension, diabetes, cholesterol levels, and total energy intake.
He	2003	40–75 (53)	Male	51,529	FFQ	NA	14	725	USA	Both	Age, BMI, physical activity, hypertension, smoking, aspirin, multivitamins, alcohol, potassium, fiber, vitamin E, fruit and vegetables, total energy intake, and hypercholesterolaemia at baseline. Additional adjustments based on other fat categories.
Iso	2003	40–69 (< 60)	Both	4,775	24-hour recall	< 24	14.3	295	Japan	Both	Age, sex, total energy intake and BMI, hypertension, diabetes, serum cholesterol, smoking, ethanol intake, and menopausal status.
Sauvaget	2004	35–89 (< 60)	Both	3,731	24-hour recall	< 24	14	90	Japan	Fatal	Sex and age, radiation dose, city, BMI, smoking, alcohol, hypertension and diabetes.
Larsson	2012	49–83 (≥ 60)	Female	34,670	FFQ	≥ 24	10.4	1,680	Sweden	Both	Age, smoking, education, BMI, physical activity, hypertension, diabetes, aspirin use, myocardial infarction, alcohol, protein, dietary fiber, specific types of fat and cholesterol.
Yaemsiri	2012	50–79 (≥ 60)	Female	87,025	FFQ	≥ 24	7.6	1,049	USA	Both	Age, race, education, income, smoking, hormone use, total metabolic equivalent task hours per week, alcohol, coronary heart disease, atrial fibrillation, diabetes, aspirin use, use of antihypertensive medication, use of cholesterol-lowering medication, BMI, SBP, and energy, dietary vitamin E, fruits, vegetable, and fiber.

SBP, systolic blood pressure; BMI, body mass index; DBP, diastolic blood pressure; FFQ, frequent food questionnaire; NA, not available.

### Cholesterol intake and stroke risk

**Figure 2:**
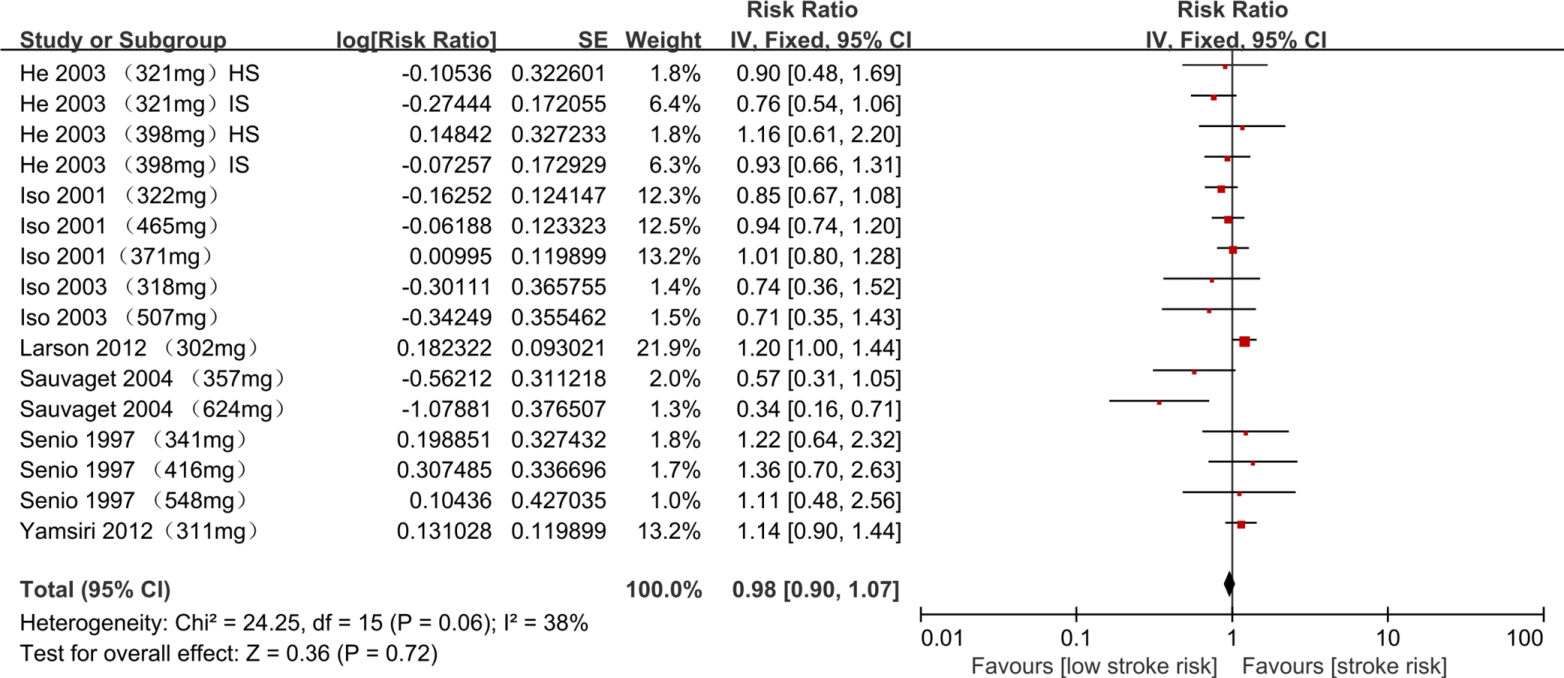
Meta-analysis of relative risks for cholesterol intake and overall stroke risk RR, relative risk; IS, ischemic stroke; HS, hemorrhagic stroke. The RRs of total, ischemic and hemorrhagic stroke for Iso et al. (2001) were retrieved from the supplemental data.

The combined RR of stroke for the highest versus lowest dietary cholesterol intake was 0.98 (95% CI, 0.90–1.07) (overall stroke risk) (Figure 2).

### Subgroup and sensitivity analyses

**Figure 3:**
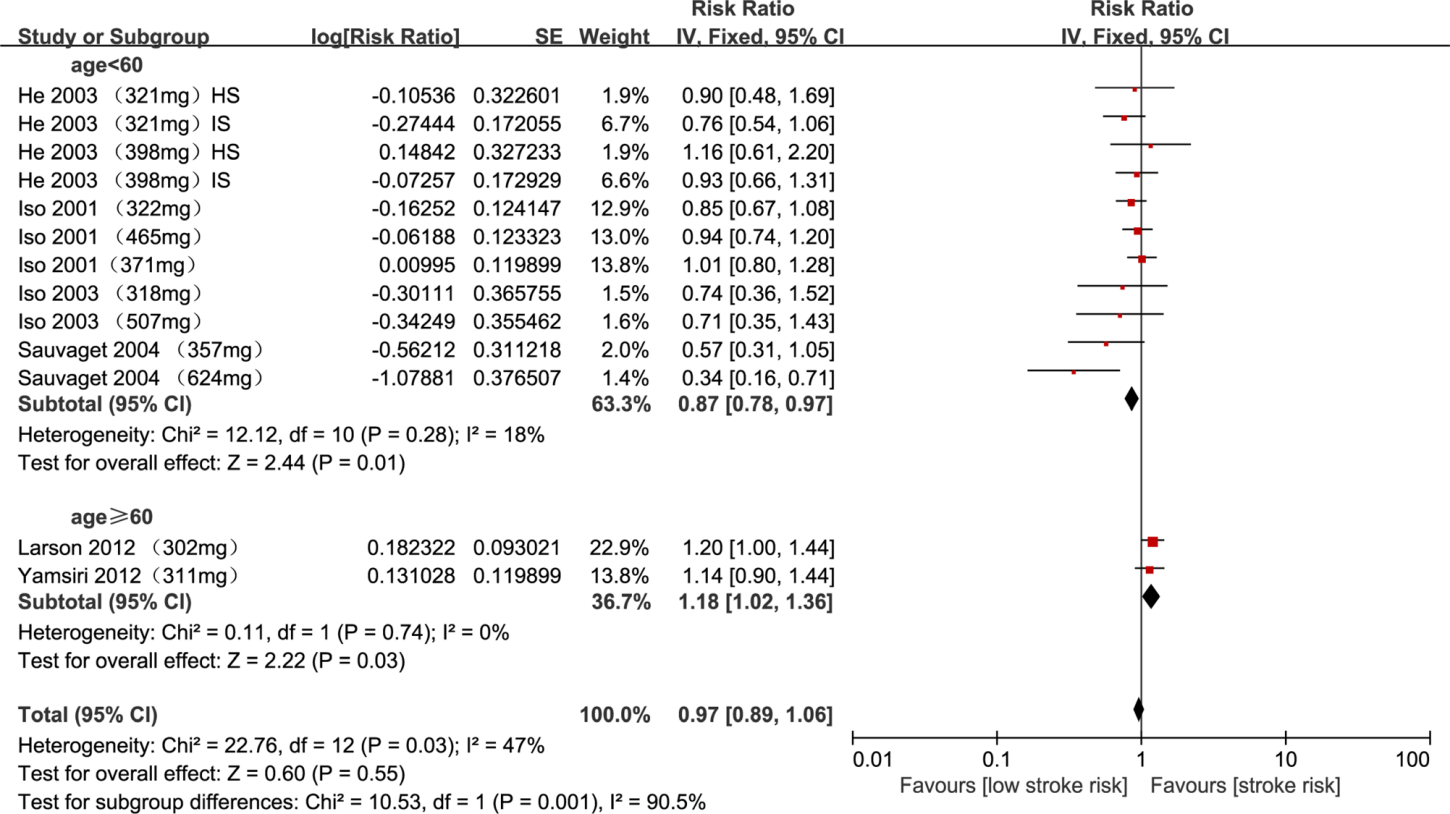
Meta-analysis of relative risks for cholesterol intake in sex subgroups RR, relative risk; IS, ischemic stroke; HS, hemorrhagic stroke.

**Figure 4:**
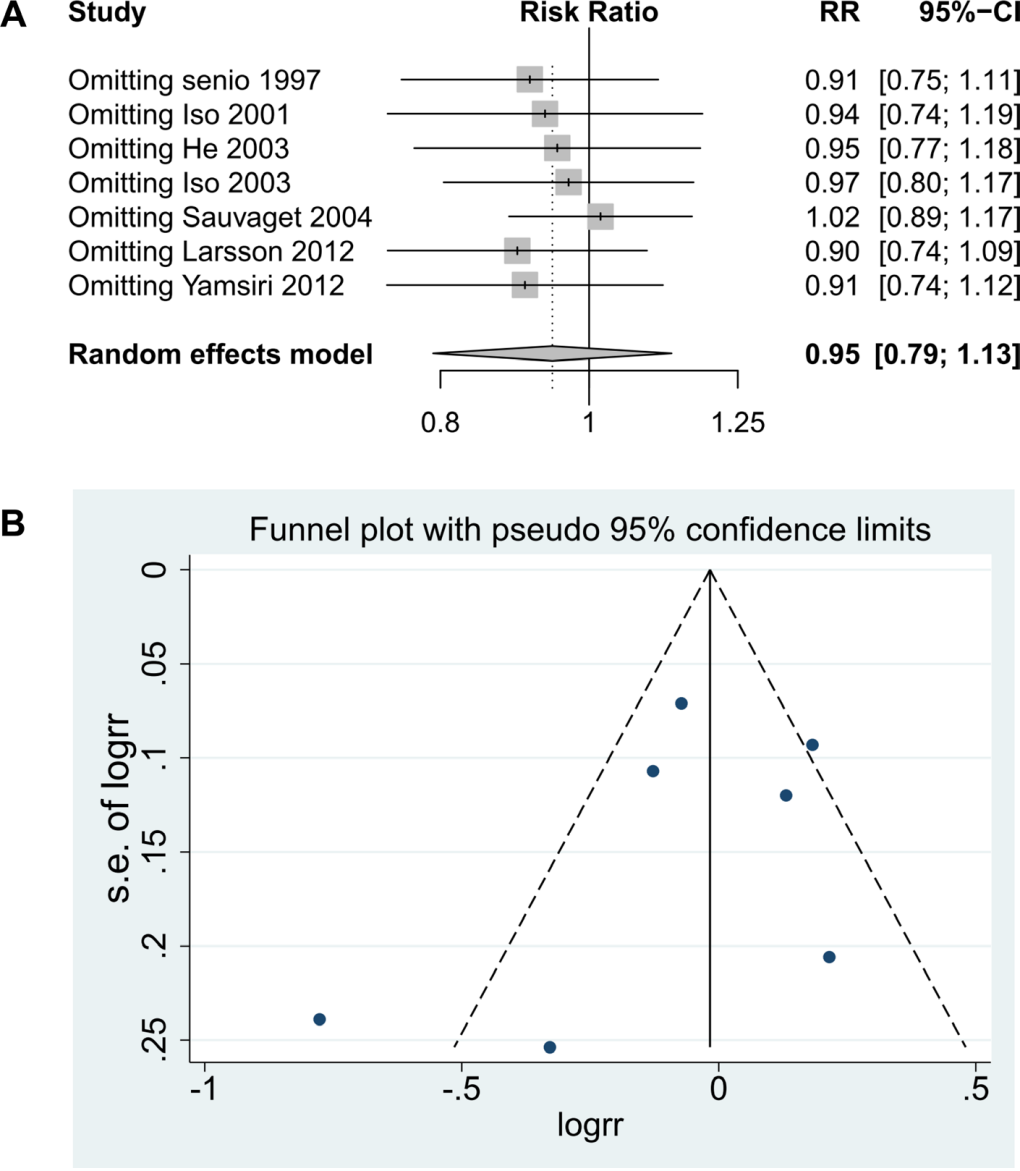
Funnel plot for cholesterol intake and stroke risk

Table 2 shows that the combined RRs of stroke for high cholesterol intake were 0.87 (95% CI, 0.78–0.97) for the < 60-year age subgroup and 1.18 (95% CI, 1.02–1.36) for the ≥ 60-year age subgroup (Figure 3). The combined RR of stroke for high cholesterol intake was 1.18 (95% CI, 1.02–1.36) for females (age of ≥ 60 years, BMI of ≥ 24 kg/m^2^, or follow-up duration of < 14 years). The association between cholesterol intake and stroke risk did not differ substantially by stroke type, sex, stroke risk for females, age, age for females, follow-up duration, ethnicity, country, study quality, or caloric intake. To determine the impact of multivariable adjustment, we performed additional sensitivity analyses by excluding studies that did not simultaneously adjust for hypertension, diabetes, and smoking. The sensitivity analyses did not lead to any change in the significance or direction of effect for the association between cholesterol intake and stroke risk after applying the leave-one-out method (Figure 4A).

**Table 2:** Stratification analysis of dietary cholesterol intake and stroke risk

				Heterogeneity	
Group	No. of studies	RR (95% CI)	X^2^	*P* value	*I*^2^ (%)
Overall stroke	7	0.98 (0.90–1.07)	24.25	0.06	38
Stroke type					
Ischemic	6	0.95 (0.80–1.12)	24.91	0.009	55.8
Hemorrhagic	4	1.03 (0.85–1.25)	4.96	0.894	0
Sex					
Both	3	0.79 (0.61–1.03)	11.23	0.081	46.6
Female	3	1.04 (0.94–1.15)	6.32	0.176	36.7
Male	1	0.88 (0.71–1.08)	-	-	-
Stroke risk for females					
Ischemic	3	1.09 (0.97–1.23)	7.46	0.113	46.4
Hemorrhagic	2	1.12 (0.89–1.42)	2.24	0.896	0
Age					
< 60 years	4	0.87 (0.78–0.97)	12.12	0.277	17.5
≥ 60 years	2	1.18 (1.02–1.36)	0.11	0.735	0
Age for females					
< 60 years	1	1.37 (0.88–2.13)	NA	NA	NA
≥ 60 years	2	1.18 (1.02–1.36)	0.11	0.735	0
Follow up duration					
< 14 years	2	1.18 (1.02–1.36)	0.11	0.735	0
≥ 14 years	5	0.89 (0.80–0.99)	15.03	0.306	13.5
Race/Ethnicity					
East-Asians	3	0.79 (0.61–1.03)	11.23	0.081	46.6
Non East-Asians	4	1.01 (0.92–1.11)	9.99	0.266	19.9
Fatal stroke risk	1	0.46 (0.29–0.74)	-	-	-
Maximum multivariates	6	0.97 (0.89–1.06)	22.76	0.030	47.3
BMI					
< 24	3	0.79 (0.61–1.03)	11.23	0.081	46.6
≥ 24	2	1.18 (1.02–1.36)	0.11	0.735	0
BMI for females					
< 24	0	NA	NA	NA	NA
≥ 24	2	1.18 (1.02–1.36)	0.11	0.735	0
Country					
USA	3	0.96 (0.86–1.06)	5.47	0.603	0
Japan	3	0.79 (0.61–1.03)	11.23	0.081	46.6
Sweden	1	1.20 (1.00–1.44)	-	-	-
Quality					
Score > 8	2	1.05 (0.91–1.20)	6.4	0.171	37.5
Score ≤ 8	5	0.95 (0.85–1.05)	16.51	0.086	39.4
Caloric intake controlled					
Yes	5	0.96 (0.87–1.06)	8.42	0.751	0
No	2	1.06 (0.89–1.26)	14.87	0.001	86.6

Abbreviations: NA, not available; BMI, body mass index.

### Meta-regression analysis

We performed a meta-regression analysis to explore the potential sources of heterogeneity. We found that race, country, stroke type, age, study quality, follow up duration, sex, BMI, or caloric intake could alone could not explain the sources of heterogeneity (*p* > 0.05).

### Publication bias

No publication bias was found for cholesterol intake and stroke risk by Egger’s test (*p* = 0.441) or by visual inspection of symmetry (Figure 4B).

## DISCUSSION

This meta-analysis included 7 prospective cohort studies [16–22] involving 269,777 individuals and demonstrated that high dietary cholesterol intake is not associated with the overall stroke risk. In the past two decades, the role of dietary cholesterol in the development of stroke has been increasingly recognized. Some studies [12, 23] have proven that dietary cholesterol intake has a positive relationship with blood pressure. However, the results of all seven prospective cohort studies in the present meta-analysis remained inconsistent. The results of five of the seven included prospective studies [16–18, 21, 22] did not support an association between cholesterol intake and stroke risk, one study [20] suggested that high animal fat and cholesterol intake was associated with a reduced risk of cerebral infarction-related death, and another study [19] suggested that dietary cholesterol intake was positively associated with stroke risk. Although a previous study [24] demonstrated that dietary cholesterol was not associated with the risk of stroke, this could be well explained by the fact that two additional cohort studies were added to our meta-analysis, and the results were re-estimated based on a much higher number of participants. The novelty of our study lies in the fact that the studies included in this meta-analysis reported cholesterol intake with different measurement units (e.g., grams, servings, or tertiles, quartiles, and quintile), and because grams of cholesterol intake varied among the studies, we demarcated cholesterol intake of > 300 mg/day as the high category for each study to ensure the credibility of the results. In addition, we estimated the relationship between dietary cholesterol and stroke risk by more subgroups such as sex, age, stroke risk for females, BMI, and others.

The results of the stratification analysis suggest that higher dietary cholesterol intake appears to raise the risk of stroke in older individuals. The increment of dietary cholesterol intake is associated with the elevation of serum cholesterol concentration [25, 26]. Consist with our previous studies [5, 27–31], lipoproteins were found to be abnormal in peripheral body fluids of some patients with neuropsychiatric disorders. We also found disturbances in lipid metabolism of central nervous system in animal models of neuropsychiatric disorders [32–34]. Cholesterol supplementation can increase the levels of total cholesterol, low-density lipoprotein (LDL) cholesterol, and high-density lipoprotein (HDL) cholesterol [35, 36], and evidence has shown that a higher cholesterol concentration is associated with a risk of ischemic cerebrovascular disease [37, 38]. Higher cholesterol intake can increase the degree of lipid peroxidation, which is one of the early stages of atherosclerosis [39–41]. Additionally, higher cholesterol intake upregulates the serum concentration of N^G^-dimethylarginine, which is thought to contribute to the development of atherosclerosis [42]. Our stratification results also demonstrated that higher cholesterol intake increases the stroke risk for older females and females with a higher BMI. Evidence shows that clearance of lipemia after intake of fat deteriorates with age [43]. The BMI is another important risk factor influencing stroke risk [44–46], and physical activity has been proven to be efficient in the treatment of elevated LDL cholesterol levels [47]. However, the results demonstrated no associations between cholesterol intake and stroke risk in other subgroups such as stroke type, sex, stroke risk for females, age, age for females, follow-up duration, ethnicity, country, study quality, and caloric intake. In addition, the sensitivity analyses led to no change in the significance or direction of effect for the association between cholesterol intake and stroke risk after applying the leave-one-out method.

We performed a meta-regression analysis to detect the sources of heterogeneity, but no sources of heterogeneity were identified. However, the heterogeneity among studies decreased in both the age and follow-up duration subgroups while analyzing the associations between cholesterol intake and stroke risk, which may partly explain the sources of heterogeneity. The funnel plot was basically symmetrical by visual inspection, and Egger’s test demonstrated no publication bias.

This study has several limitations. First, only one study [20] provided fatal stroke risk data for dietary cholesterol intake; thus, the fatal stroke risk with respect to cholesterol intake could not be assessed in this study. Future studies should be performed to investigate the associations between fatal stroke risk and cholesterol intake. Second, the possibility that other factors may have affected the observed association cannot be excluded. For instance, based on our results, there seems to be a positive association between cholesterol intake and risk of stroke in females aged > 60 years. Women, particularly of this age group, have reached menopause and thus have an altered lipoprotein profile because of their low estrogen concentration. Thus, even if their diet is not high in cholesterol, they still have a slightly abnormal lipid profile [48]. Our data should therefore be interpreted with caution. Dietary potassium, magnesium, fiber, and protein are factors [49–52] influencing dietary cholesterol intake and risk of stroke. However, when we confined the maximum multivariates analysis to the included studies, there remained no association between dietary cholesterol intake and stroke risk. Third, this meta-analysis included both East Asians and non-East Asians. The upper limit of a normal BMI for Asian populations should be 23 kg/m^2^, and the 1997 World Health Organization guidelines specify that the upper limit of a normal BMI was 25 kg/m^2^ [53]. Thus, we used a midpoint BMI of 24 kg/m^2^ to divide the subgroups and minimize the bias of the observational results. Fourth, only one study [16] in this meta-analysis reported the association between dietary cholesterol intake and stroke risk in males, preventing evaluation of the pooled RRs of stroke for cholesterol intake in males. Fifth, familial hypercholesterolemia is an important factor that contributes to the incidence of stroke [54]. However, only one study [16] in this meta-analysis reported RRs with control for hypercholesterolemia at baseline. Future studies should report RRs for the association between cholesterol intake and stroke risk with control for this factor. Sixth, no data regarding changes in the serum lipid profile (i.e., LDL, VLDL and HDL cholesterol levels) were available in the studies of this meta-analysis; dietary cholesterol intake of LDL alone is known to be involved in the pathogenesis of atherosclerosis and HDL cholesterol is considered to be cardio protective.

In summary, higher cholesterol intake has no association with the overall stroke risk. Age and BMI affect the relationship between dietary cholesterol intake and stroke risk. However, the association between high dietary cholesterol intake and stroke risk in males remains unclear.

## MATERIALS AND METHODS

### Literature search strategy

We searched PubMed, Medline, Embase, Web of Knowledge, and Google Scholar without language restrictions using the following keywords: (“cholesterol”) combined with (“stroke” OR “cerebrovascular disease” OR “cerebrovascular disorder” OR “cerebrovascular accident”). Other potential articles (if any) were identified by consulting previous reviews and reference lists of retrieved records.

### Inclusion and exclusion criteria

We excluded letters, comments, reviews, meta-analyses, ecological studies, and experimental studies. Studies were included if they were cohort studies, involved more than 2,000 participants, studied the effects of specific levels of cholesterol intake, provided details regarding cholesterol intake in grams, provided relative risks (RRs) and 95% confidence intervals (CIs), and reported the risk of all types of stroke ischemic stroke, hemorrhagic stroke, or fatal stroke as the outcome of interest. Potentially relevant studies were independently identified by two investigators (F.P.C. and W.H.); any discrepancies were resolved by consensus or consultation with a third author (P.X.). We used the Newcastle–Ottawa Scale [55] to assess the quality of the studies in this meta-analysis.

### Data extraction

Data were independently extracted by two investigators (P.F.C. and W.H.), and differences were resolved by discussion with a third investigator (S.J.B.). We retrieved relevant epidemiological data; the first author’s name; the publication year; the country of the study population; the age, sex, and number of participants; assessment of cholesterol intake; BMI; follow-up duration; number of stroke events; outcome assessment (fatal or nonfatal); RRs of stroke and corresponding 95% CIs for cholesterol intake; and covariates adjusted in the statistical analysis.

### Statistical analysis

The studies included in this meta-analysis reported cholesterol intake with different measurement units (e.g., grams, servings, or tertiles, quartiles, and quintiles), and because the grams of cholesterol intake varied among studies, we demarcated a cholesterol intake of > 300 mg [56] per day as the high category for each study. The summary RR was combined using the inverse variance method for this meta-analysis, and the 95% CI was used to interpret the results. An I^2^ of 25%, 50%, and 75% represented low, moderate, and high heterogeneity, respectively. We used a fixed-effects model when I^2^ < 50%; otherwise, we used a random-effects model to calculate the pooled RRs of stroke for dietary cholesterol intake. We retrieved all supplemental files of the included studies for the RRs of specific stroke types, if available.

Subgroup analyses were conducted based on the following 14 stratifications: stroke type, sex, stroke risk for females, age, age for females, follow-up duration, ethnicity, fatal stroke risk, maximum multivariates (hypertension, diabetes, and smoking adjusted simultaneously because these 3 factors are closely related to stroke risk [57–60]), BMI, BMI for females, country, study quality, and caloric intake. Hypertension, diabetes, and smoking were not controlled simultaneously in one study [21], and this study did not explicitly exclude individuals with diabetes or smoking; therefore, we did not include this study in the maximum multivariate-adjusted analysis. We evaluated the heterogeneity among the studies with the Q and I^2^ statistics [61]. The sensitivity analyses for the association between cholesterol intake and stroke risk were conducted by applying the leave-one-out method. Egger’s test was used to check for any particular publication biases and their magnitude, and a funnel plot was prepared for visual inspection of symmetry. Data analyses were conducted with the Stata software package (version 12.0; StataCorp, College Station, TX, USA) and Review Manager (RevMan) 5.3 software (version 5.3.5; The Nordic Cochrane Centre, The Cochrane Collaboration, Copenhagen, Denmark).

## SUPPLEMENTARY MATERIALS TABLE



## References

[1] Zhang L, Wu J, Duan X, Tian X, Shen H, Sun Q, Chen G (2016). NADPH Oxidase: A Potential Target for Treatment of Stroke. Oxid Med Cell Longev.

[2] Cheng P, Huang W, Bai S, Wu Y, Yu J, Zhu X, Qi Z, Shao W, Xie P (2015). BMI Affects the Relationship between Long Chain N-3 Polyunsaturated Fatty Acid Intake and Stroke Risk: a Meta-Analysis. Sci Rep.

[3] Wang T, Li B, Gu H, Lou Y, Ning X, Wang J, An Z (2017). Effect of age on long-term outcomes after stroke with atrial fibrillation: a hospital-based follow-up study in China. Oncotarget.

[4] Pan J, Liu H, Zhou J, Liu Z, Yang Y, Peng Y, You H, Yang D, Xie P (2014). Ipsilateral hippocampal proteomics reveals mitochondrial antioxidative stress impairment in cortical-lesioned chronic mild stressed rats. Curr Mol Med.

[5] Zhan Y, Yang YT, You HM, Cao D, Liu CY, Zhou CJ, Wang ZY, Bai SJ, Mu J, Wu B, Zhan QL, Xie P (2014). Plasma-based proteomics reveals lipid metabolic and immunoregulatory dysregulation in post-stroke depression. Eur Psychiatry.

[6] Feigin VL, Forouzanfar MH, Krishnamurthi R, Mensah GA, Connor M, Bennett DA, Moran AE, Sacco RL, Anderson L, Truelsen T, O’Donnell M, Venketasubramanian N, Barker-Collo S (2014). Global Burden of Deiseases, Injuries, and Risk Factors Study 2010 (GBD 2010); GBD Stroke Experts Group. Global and regional burden of stroke during 1990–2010: findings from the Global Burden of Disease Study 2010. Lancet.

[7] Kahles T, Brandes RP (2013). Which NADPH oxidase isoform is relevant for ischemic stroke? The case for nox 2. Antioxid Redox Signal.

[8] Chen GC, Lv DB, Pang Z, Dong JY, Liu QF (2013). Dietary fiber intake and stroke risk: a meta-analysis of prospective cohort studies. Eur J Clin Nutr.

[9] Strong K, Mathers C, Bonita R (2007). Preventing stroke: saving lives around the world. Lancet Neurol.

[10] Lim H, Choue R (2013). Impact of nutritional status and dietary quality on stroke: do we need specific recommendations?. Eur J Clin Nutr.

[11] Bradbury KE, Crowe FL, Appleby PN, Schmidt JA, Travis RC, Key TJ (2015). Serum concentrations of cholesterol, apolipoprotein A-I and apolipoprotein B in a total of 1694 meat-eaters, fish-eaters, vegetarians and vegans. Eur J Clin Nutr.

[12] Stamler J, Caggiula A, Grandits GA, Kjelsberg M, Cutler JA (1996). Relationship to blood pressure of combinations of dietary macronutrients. Findings of the Multiple Risk Factor Intervention Trial (MRFIT). Circulation.

[13] Threapleton DE, Burley VJ, Greenwood DC, Cade JE (2015). Dietary fibre intake and risk of ischaemic and haemorrhagic stroke in the UK Women's Cohort Study. Eur J Clin Nutr.

[14] Staessen JA, Gasowski J, Wang JG, Thijs L, Den Hond E, Boissel JP, Coope J, Ekbom T, Gueyffier F, Liu L, Kerlikowske K, Pocock S, Fagard RH (2000). Risks of untreated and treated isolated systolic hypertension in the elderly: meta-analysis of outcome trials. Lancet.

[15] Rubattu S, Bianchi F, Busceti CL, Cotugno M, Stanzione R, Marchitti S, Di Castro S, Madonna M, Nicoletti F, Volpe M (2015). Differential modulation of AMPK/PPARalpha/UCP2 axis in relation to hypertension and aging in the brain, kidneys and heart of two closely related spontaneously hypertensive rat strains. Oncotarget.

[16] He K, Merchant A, Rimm EB, Rosner BA, Stampfer MJ, Willett WC, Ascherio A (2003). Dietary fat intake and risk of stroke in male US healthcare professionals: 14 year prospective cohort study. BMJ.

[17] Iso H, Sato S, Kitamura A, Naito Y, Shimamoto T, Komachi Y (2003). Fat and protein intakes and risk of intraparenchymal hemorrhage among middle-aged Japanese. Am J Epidemiol.

[18] Iso H, Stampfer MJ, Manson JE, Rexrode K, Hu F, Hennekens CH, Colditz GA, Speizer FE, Willett WC (2001). Prospective study of fat and protein intake and risk of intraparenchymal hemorrhage in women. Circulation.

[19] Larsson SC, Virtamo J, Wolk A (2012). Dietary fats and dietary cholesterol and risk of stroke in women. Atherosclerosis.

[20] Sauvaget C, Nagano J, Hayashi M, Yamada M (2004). Animal protein, animal fat, and cholesterol intakes and risk of cerebral infarction mortality in the adult health study. Stroke.

[21] Seino F, Date C, Nakayama T, Yoshiike N, Yokoyama T, Yamaguchi M, Tanaka H (1997). Dietary lipids and incidence of cerebral infarction in a Japanese rural community. J Nutr Sci Vitaminol (Tokyo).

[22] Yaemsiri S, Sen S, Tinker L, Rosamond W, Wassertheil-Smoller S, He K (2012). Trans fat, aspirin, and ischemic stroke in postmenopausal women. Ann Neurol.

[23] Vikdahl M, Backman L, Johansson I, Forsgren L, Haglin L (2015). Cardiovascular risk factors and the risk of Parkinson's disease. Eur J Clin Nutr.

[24] Berger S, Raman G, Vishwanathan R, Jacques PF, Johnson EJ (2015). Dietary cholesterol and cardiovascular disease: a systematic review and meta-analysis. Am J Clin Nutr.

[25] Hopkins PN (1992). Effects of dietary cholesterol on serum cholesterol: a meta-analysis and review. Am J Clin Nutr.

[26] Chen KC, Liao YC, Wang JY, Lin YC, Chen CH, Juo SH (2015). Oxidized low-density lipoprotein is a common risk factor for cardiovascular diseases and gastroenterological cancers via epigenomical regulation of microRNA-210. Oncotarget.

[27] Xu HB, Zhang RF, Luo D, Zhou Y, Wang Y, Fang L, Li WJ, Mu J, Zhang L, Zhang Y, Xie P (2012). Comparative proteomic analysis of plasma from major depressive patients: identification of proteins associated with lipid metabolism and immunoregulation. Int J Neuropsychopharmacol.

[28] Zheng P, Gao HC, Li Q, Shao WH, Zhang ML, Cheng K, Yang DY, Fan SH, Chen L, Fang L, Xie P (2012). Plasma metabonomics as a novel diagnostic approach for major depressive disorder. J Proteome Res.

[29] Mu J, Yang Y, Chen J, Cheng K, Li Q, Wei Y, Zhu D, Shao W, Zheng P, Xie P (2015). Elevated host lipid metabolism revealed by iTRAQ-based quantitative proteomic analysis of cerebrospinal fluid of tuberculous meningitis patients. Biochem Biophys Res Commun.

[30] Song YR, Wu B, Yang YT, Chen J, Zhang LJ, Zhang ZW, Shi HY, Huang CL, Pan JX, Xie P (2015). Specific alterations in plasma proteins during depressed, manic, and euthymic states of bipolar disorder. Braz J Med Biol Res.

[31] Liu X, Zheng P, Zhao X, Zhang Y, Hu C, Li J, Zhao J, Zhou J, Xie P, Xu G (2015). Discovery and validation of plasma biomarkers for major depressive disorder classification based on liquid chromatography-mass spectrometry. J Proteome Res.

[32] Liu L, Zhou X, Zhang Y, Liu Y, Yang L, Pu J, Zhu D, Zhou C, Xie P (2016). The identification of metabolic disturbances in the prefrontal cortex of the chronic restraint stress rat model of depression. Behav Brain Res.

[33] Wu Y, Fu Y, Rao C, Li W, Liang Z, Zhou C, Shen P, Cheng P, Zeng L, Zhu D, Zhao L, Xie P (2016). Metabolomic analysis reveals metabolic disturbances in the prefrontal cortex of the lipopolysaccharide-induced mouse model of depression. Behav Brain Res.

[34] Wu Y, Tang J, Zhou C, Zhao L, Chen J, Zeng L, Rao C, Shi H, Liao L, Liang Z, Yang Y, Zhou J, Xie P (2016). Quantitative proteomics analysis of the liver reveals immune regulation and lipid metabolism dysregulation in a mouse model of depression. Behav Brain Res.

[35] Faghihnia N, Mangravite LM, Chiu S, Bergeron N, Krauss RM (2012). Effects of dietary saturated fat on LDL subclasses and apolipoprotein CIII in men. Eur J Clin Nutr.

[36] Clifton PM, Nestel PJ (1992). Influence of gender, body mass index, and age on response of plasma lipids to dietary fat plus cholesterol. Arterioscler Thromb.

[37] Lindenstrom E, Boysen G, Nyboe J (1994). Influence of total cholesterol, high density lipoprotein cholesterol, and triglycerides on risk of cerebrovascular disease: the Copenhagen City Heart Study. BMJ.

[38] Zhang Y, Bai L, Shi M, Lu H, Wu Y, Tu J, Ni J, Wang J, Cao L, Lei P, Ning X (2017). Features and risk factors of carotid atherosclerosis in a population with high stroke incidence in China. Oncotarget.

[39] Hoebeeck LI, Rietzschel ER, Langlois M, De Buyzere M, De Bacquer D, De Backer G, Maes L, Gillebert T, Huybrechts I (2011). The relationship between diet and subclinical atherosclerosis: results from the Asklepios Study. Eur J Clin Nutr.

[40] Jeon SM, Bok SH, Jang MK, Lee MK, Nam KT, Park YB, Rhee SJ, Choi MS (2001). Antioxidative activity of naringin and lovastatin in high cholesterol-fed rabbits. Life Sci.

[41] Tsai KL, Hung CH, Chan SH, Shih JY, Cheng YH, Tsai YJ, Lin HC, Chu PM (2016). Baicalein protects against oxLDL-caused oxidative stress and inflammation by modulation of AMPK-alpha. Oncotarget.

[42] Yu XJ, Li YJ, Xiong Y (1994). Increase of an endogenous inhibitor of nitric oxide synthesis in serum of high cholesterol fed rabbits. Life Sci.

[43] Cohn JS, McNamara JR, Cohn SD, Ordovas JM, Schaefer EJ (1988). Postprandial plasma lipoprotein changes in human subjects of different ages. J Lipid Res.

[44] Kurth T, Gaziano JM, Berger K, Kase CS, Rexrode KM, Cook NR, Buring JE, Manson JE (2002). Body mass index and the risk of stroke in men. Arch Intern Med.

[45] Song YM, Sung J, Davey Smith G, Ebrahim S (2004). Body mass index and ischemic and hemorrhagic stroke: a prospective study in Korean men. Stroke.

[46] Strazzullo P, D'Elia L, Cairella G, Garbagnati F, Cappuccio FP, Scalfi L (2010). Excess body weight and incidence of stroke: meta-analysis of prospective studies with 2 million participants. Stroke.

[47] Stefanick ML, Mackey S, Sheehan M, Ellsworth N, Haskell WL, Wood PD (1998). Effects of diet and exercise in men and postmenopausal women with low levels of HDL cholesterol and high levels of LDL cholesterol. N Engl J Med.

[48] Gokmen O, Yapar Eyi EG (1999). Hormone replacement therapy and lipid-lipoprotein concentrations. Eur J Obstet Gynecol Reprod Biol.

[49] D'Elia L, Barba G, Cappuccio FP, Strazzullo P (2011). Potassium Intake, Stroke, and Cardiovascular DiseaseA Meta-Analysis of Prospective Studies. J Am Coll Cardiol.

[50] Larsson SC, Orsini N, Wolk A (2012). Dietary magnesium intake and risk of stroke: a meta-analysis of prospective studies. Am J Clin Nutr.

[51] Zhang Z, Xu G, Liu D, Zhu W, Fan X, Liu X (2013). Dietary fiber consumption and risk of stroke. Eur J Epidemiol.

[52] Zhang Z, Xu G, Yang F, Zhu W, Liu X (2014). Quantitative analysis of dietary protein intake and stroke risk. Neurology.

[53] Choo V (2002). WHO reassesses appropriate body-mass index for Asian populations. Lancet.

[54] Hutter CM, Austin MA, Humphries SE (2004). Familial hypercholesterolemia, peripheral arterial disease, and stroke: a HuGE minireview. Am J Epidemiol.

[55] Stang A (2010). Critical evaluation of the Newcastle-Ottawa scale for the assessment of the quality of nonrandomized studies in meta-analyses. Eur J Epidemiol.

[56] Lichtenstein AH, Appel LJ, Brands M, Carnethon M, Daniels S, Franch HA, Franklin B, Kris-Etherton P, Harris WS, Howard B, Karanja N, Lefevre M, Rudel L, American Heart Association Nutrition Committee (2006). Diet and lifestyle recommendations revision 2006: a scientific statement from the American Heart Association Nutrition Committee. Circulation.

[57] Wolf PA, D'Agostino RB, Kannel WB, Bonita R, Belanger AJ (1988). Cigarette smoking as a risk factor for stroke: the Framingham Study. JAMA.

[58] Shinton R, Beevers G (1989). Meta-analysis of relation between cigarette smoking and stroke. BMJ.

[59] Wolf PA, D'Agostino RB, Belanger AJ, Kannel WB (1991). Probability of stroke: a risk profile from the Framingham Study. Stroke.

[60] Peters SA, Huxley RR, Woodward M (2014). Diabetes as a risk factor for stroke in women compared with men: a systematic review and meta-analysis of 64 cohorts, including 775,385 individuals and 12,539 strokes. The Lancet.

[61] Higgins J, Thompson SG (2002). Quantifying heterogeneity in a meta-analysis. Stat Med.

