# Correction: Dietary cholesterol intake and stroke risk: a meta-analysis

**DOI:** 10.18632/oncotarget.25644

**Published:** 2018-06-12

**Authors:** Pengfei Cheng, Junxi Pan, Jinjun Xia, Wen Huang, Shunjie Bai, Xiaofeng Zhu, Weihua Shao, Haiyang Wang, Peng Xie

**Affiliations:** ^1^ Department of Neurology, The First Affiliated Hospital of Chongqing Medical University, Chongqing, 400016, China; ^2^ Chongqing Key Laboratory of Neurobiology, Chongqing, 400016, China; ^3^ Institute of Neuroscience and The Collaborative Innovation Center for Brain Science, Chongqing Medical University, Chongqing, 400016, China; ^4^ Department of Neurology, The First Affiliated Hospital of Jiamusi University, Jiamusi, Heilongjiang Province, 154002, China; ^5^ The M.O.E. Key Laboratory of Laboratory Medical Diagnostics, The College of Laboratory Medicine, Chongqing Medical University, Chongqing, 400016, China; ^6^ Department of Neurology, Xinqiao Hospital, Third Military Medical University, Chongqing 400037, China; ^7^ Institute of Neuroscience, Jiamusi University, Jiamusi, Heilongjiang Province, 154002, China; ^8^ Department of Respiratory Medicine, The First Affiliated Hospital, Chongqing Medical University, Chongqing, 400016, China

**This article has been corrected:** The correct author information is given below:

Pengfei Cheng^1,2,3,4,*^, Junxi Pan^2,3,5,*^, Jinjun Xia^2,3,5,*^, Wen Huang^6^, Shunjie Bai^2,3,5^, Xiaofeng Zhu^7^, Weihua Shao^8^, Haiyang Wang^2,3^ and Peng Xie^1,2,3^

Original article: Oncotarget. 2018; 9:25698-25707. https://doi.org/10.18632/oncotarget.23933

