# Supplementary material for: Dietary cholesterol intake and stroke risk: a meta-analysis
Source: Oncotarget. 2018 Jan 4;9(39):25698–707. doi: 10.18632/oncotarget.23933 (PMC5986647; doi:10.18632/oncotarget.23933)
Supplement: Supplementary file 1 [file oncotarget-09-25698-s001.pdf]

Dietary cholesterol intake and stroke risk: a meta-analysis

SUPPLEMENTARY MATERIALS

Supplementary Table 1: Newcastle - Ottawa quality assessment scale of the included studies

Study	Selection				Comparability	Exposure			Score
	CDA	RC	SC	DC		AE	SMACC	NRR	
**Seino 1997**	1	0	1	1	2	1	1	1	8
**Iso 2001**	1	0	1	1	2	1	1	1	8
**He 2003**	1	1	1	1	2	1	1	1	9
**Iso 2003**	1	1	1	1	2	0	1	1	8
**Sauvaget 2004**	1	0	0	1	2	1	1	1	7
**Larsson 2012**	1	1	1	1	2	1	1	1	9
**Yaemsiri 2012**	1	1	1	1	2	0	1	1	8

Abbreviations: CDA, case definition adequate; RC, Representativeness of the cases; SC, selection of controls; DC, definition of controls; AE, ascertainment of exposure; SMACC, same method of ascertainment for cases and controls; NRR, non-response rates.
